# Dual-Port Six-Band Rectenna with Enhanced Power Conversion Efficiency at Ultra-Low Input Power

**DOI:** 10.3390/s24237433

**Published:** 2024-11-21

**Authors:** Shihao Sun, Yuchao Wang, Bingyang Li, Hanyu Xue, Cheng Zhang, Feng Xu, Chaoyun Song

**Affiliations:** 1 School of Physics and Mechanics, Wuhan University of Technology, Wuhan 430070, China; shihao_sun@whut.edu.cn (S.S.); yuchao9629@whut.edu.cn (Y.W.); xhy1003@whut.edu.cn (H.X.); 2 China Academy of Aerospace Science and Innovation, Beijing 100094, China; libingyang@stu.pku.edu.cn; 3 Shanghai Institute of Optics and Fine Mechanics, Chinese Academy of Sciences, Shanghai 201800, China; czhang2024@siom.ac.cn; 4 Hangzhou Institute for Advanced Study, University of Chinese Academy of Sciences, Hangzhou 310024, China; 5 College of Electronics and Information Engineering, Shenzhen University, Shenzhen 518060, China; 6 Department of Engineering, King’s College London, London WC2R 2LS, UK

**Keywords:** RF energy harvesting, high efficiency rectennas, multi-band rectennas

## Abstract

In this paper, a novel topology and method for designing a multi-band rectenna is proposed to improve its RF-DC efficiency. The rectifier achieves simultaneous rectification using both series and parallel configurations by connecting two branches to the respective terminals of the diode, directing the energy input from two ports to the anode and cathode of the diode. Six desired operating frequency bands are evenly distributed across these two branches, each of which is connected to antennas corresponding to their specific operating frequencies, serving as the receiving end of the system. To optimize the design process, a low-pass filter is incorporated into the rectifier design. This filter works in conjunction with a matching network that includes filtering capabilities to isolate the two ports of the rectifier. The addition of the filter ensures that each structure within the rectifier can be designed independently without adversely affecting the performance of the already completed structures. Based on the proposed design methodology, a dual-port rectenna operating at six frequency bands—1.85 GHz, 2.25 GHz, 2.6 GHz, 3.52 GHz, 5.01 GHz, and 5.89 GHz—was designed, covering the 4G, 5G, and Wi-Fi/WLAN frequency bands. The measured results indicate that high-power conversion efficiency was achieved at an input power of −10 dBm: 43.01% @ 1.85 GHz, 41.00% @ 2.25 GHz, 41.33% @ 2.6 GHz, 35.88% @ 3.52 GHz, 22.36% @ 5.01 GHz, and 19.27% @ 5.89 GHz. When the input power is −20 dBm, the conversion efficiency of the rectenna can be improved from 5.2% for single-tone input to 27.7% for six-tone input, representing a 22.5 percentage point improvement. The proposed rectenna demonstrates significant potential for applications in powering low-power sensors and other devices within the Internet of Everything context.

## 1. Introduction

With the advancement of modern wireless communication technologies, particularly the introduction of Fifth-Generation Mobile Communication Technology (5G), the concept of the Internet of Things (IoT) has been further extended to the Internet of Everything (IoE). This transition signifies that a vast number of wireless devices and sensors will be interconnected through wireless communication and integrated into everyday life [1], leading to an increase in the number of these devices to 30.9 billion by 2025 [2]. Given the operational environments and the sheer volume of devices, continuously providing power to them will pose a significant challenge [2]. The resurgence of radio frequency (RF) energy harvesting (RFEH) technology in the 1990s provided an effective solution to this situation [3,4]. A rectenna represents a crucial component in an RFEH system. The rectenna is constituted of a receiving antenna and a rectifier. The receiving antenna, situated at the front end of the rectenna, is responsible for receiving RF energy from the surrounding environment. The rectifier, located at the rear end of the device, performs the conversion of this RF energy into direct current (DC) energy, which is then used to power the system’s load. The conversion efficiency of the rectenna, therefore, has a direct impact on the overall performance of the RFEH system. Compared to other energy-harvesting methods, RFEH offers significant advantages in terms of available scenarios and operational time, demonstrating the potential to provide a continuous power supply to devices [2]. This gives the rectenna great potential for IoE scenarios such as connected devices in smart homes, consumer mobile devices, and industrial sensors.

The communication frequency spectrum suitable for RFEH spans the range from 3 kHz to 300 GHz [5]. Broadband and multi-band rectennas are a better choice than single-band rectennas to take full advantage of this wide communications spectrum. However, not all frequencies in the spectrum range can be used for RFEH; only those artificially demarcated communication spectrums are better for harvesting RF energy, such as 4G LTE bands, 5G bands, etc. The discontinuous spectrum gives rise to the existence of certain invalid frequencies within the operational band range of broadband rectenna. Furthermore, there are theoretical constraints associated with the matching design of broadband rectenna [6], which ultimately results in a restricted operational bandwidth. Consequently, it is unable to effectively encompass the entire current communication spectrum with its extensive frequency range. Although the bandwidth of each operating band of the multi-band rectenna is relatively narrow, it is possible to make it work simultaneously in multiple communication bands through reasonable design, which is a more optimal choice.

In recent years, reports on multiband rectennas have categorized design methodologies into two primary approaches: matching all operational frequency bands through a complex matching network (MN) [7,8,9,10,11,12,13,14,15,16,17] and stacking multiple single-band or multiband sub-rectifiers [18,19,20,21,22,23,24]. For the first approach, as the number of frequency bands simultaneously matched by the network increases, the structure of the MN becomes increasingly complex [25]. These complex structures introduce additional insertion losses, thereby reducing the overall conversion efficiency of the rectifier. In the second approach, each sub-rectifier possesses independent rectifying diodes, and the turn-on voltage of these diodes also decreases the overall conversion efficiency of the rectifier. Consequently, for multiband rectennas, a key design challenge lies in how to achieve input across a multitude of frequency bands with a minimal number of diodes while maintaining a relatively simple structure.

**Figure 1:**
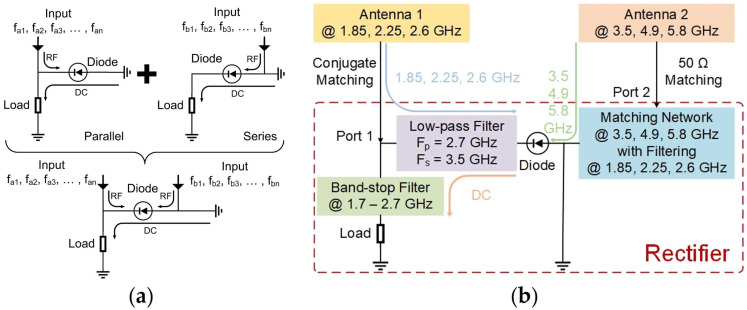
(**a**) Schematic diagram of single-diode-based dual-port rectifier formation; (**b**) overall framework of the rectenna.

In this paper, a single-diode-based dual-port six-band rectenna is proposed in Figure 1, which consists of a rectifier and two receiving antennas. As illustrated in Figure 1a, both series and parallel rectification techniques exhibit comparable load and ground positions within the circuit. However, the input ports of both are situated at the anode and cathode of the collection diode, respectively. This structure provides the foundation for the design of a single-diode two-port circuit. However, in practice, some of the RF energy input from the port will penetrate the diode without being converted to DC energy, and this needs to be limited when designing circuits for single-diode-based dual-ports. Figure 1b illustrates the framework of the rectenna. To achieve multi-band operation and high power conversion efficiency (PCE), the input impedance of antenna 1 is conjugately matched to the input impedance of the rectifier’s Port 1. Simultaneously, the input impedance of antenna 2 is adjusted to a standard 50 Ω to match Port 2 of the rectifier. Through the meticulous design of the low-pass filter (LPF) and MN, the proposed rectenna achieves high RF-DC conversion efficiencies of 43.01% at 1.85 GHz, 41.00% at 2.25 GHz, 41.33% at 2.6 GHz, 35.88% at 3.52 GHz, 22.36% at 5.01 GHz, and 19.27% at 5.89 GHz with an input power of −10 dBm. Notably, at an ultra-low input power level of −20 dBm, the rectenna’s PCE achieves significant improvement, increasing from 5.2% for single-tone input to 27.6% for six-tone input, representing an enhancement of 22.4%. These results demonstrate that the proposed rectenna performs well at low input power levels, making it suitable for RFEH applications.

## 2. Rectifier Design

To achieve single-diode rectification while minimizing insertion loss, a dual-port six-band rectifier is proposed using SMS7630 (Skyworks Solutions, Woburn, MA, USA) (turn-on voltage, V_F_ = 0.2 V, maximum reverse voltage, V_B_ = 2 V) as a rectifier diode [26], as illustrated in Figure 1. This design enables RF energy at both low and high frequencies to enter the diode through Port 1 and Port 2, respectively. It is crucial to emphasize that only RF energy entering the diode is converted to DC energy, whereas RF energy transmitted from one port to another will diminish PCE. To mitigate this issue, the RF current pathways must be pre-defined using specific microstrip-line configurations, as shown in Figure 1b. According to the required current pathway, the function of the microstrip-line configurations is as follows:

(1) Band-stop filter: For low-frequency energy, it is necessary to place a band-stop filter (BSF) in front of the load resistance to stop low-frequency energy from flowing into the load.
(2) Low-pass filter: To prevent high-frequency energy from entering the diode and being output from Port 1, a filter for high frequencies should be added to the left end of the diode. Meanwhile, the filter should achieve low insertion loss at low frequency to ensure that the low-frequency energy can pass through the filter to the diode. Therefore, an LPF needs to be designed in which the passband and stopband are set as 2.7 GHz and 3.5 GHz.
(3) Matching network with filtering function: To block low-frequency energy entering the diode from being output from Port 2, the filter for the low frequency should be added to the right end of the diode. In addition, an MN is needed to transform the input impedance of Port 2 to the standard 50 Ω. Therefore, an MN with filtering function will be designed by integrating the filter and MN.

Next, the above-mentioned configurations will be designed to ensure that RF energy from both ports is exclusively directed into the diode for conversion into DC energy.

### 2.1. Band-Stop Filter Design

**Figure 2:**
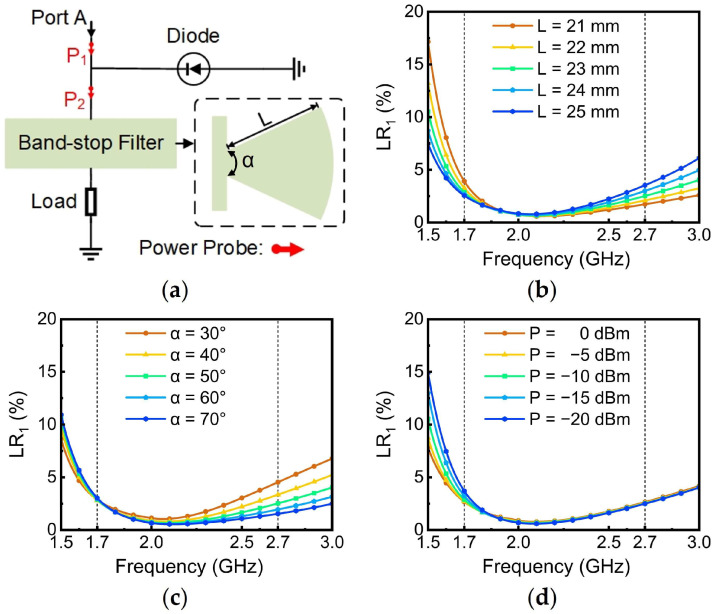
(**a**) Topological structure and performance verification circuit of the BSF; (**b**) variation in the LR_1_ within the BSF stop-band as L changes; (**c**) variation om the LR_1_ within the BSF stop-band as α changes; (**d**) variation in the LR_1_ within the BSF stop-band as input power changes.

In the topology of the rectifier, the rectifier diode and load resistor are necessary since the diode converts the RF energy into DC energy to power the load. However, directly connecting the load to the diode would inevitably cause RF energy leakage towards the load, resulting in a reduction in PCE. Therefore, a BSF should first be designed that consists of a radial stub, as shown in Figure 2a. To detect the RF current from Port A, two power probes (referred to as P1 and P2) are positioned on either side of the diode branch, as shown in Figure 2a. The probes can monitor the BSF’s ability of blocking the energy delivered to the load. The ratio of power leaking from Port A to the load, relative to the total input power, is defined as follows:

(1) $$Leakage\;Ratio_{1}=\frac{Power_{p2}}{Power_{p1}}$$

where Power_p2_ is the RF power detected by P2, and Power_p1_ is the RF power detected by P1. To achieve optimal performance of the BSF, the effect of the radial stub’s length (L) on the Leakage Ratio_1_ (LR_1_) is investigated, as shown in Figure 2b. From Figure 2b, it can be observed that as L progressively increases, the leakage ratio of the BSF gradually decreases at 1.7 GHz, while it rapidly increases at 2.7 GHz. By balancing the filter performance across the frequency range of 1.7–2.7 GHz, 23 mm is determined to be the optimal value for L. Moreover, as shown in Figure 2c, it can be observed that increasing the angle (α) of the radial stub leads to a decrease in the leakage ratio. It is important to note that a larger angle of the radial stub results in an increased size of the rectifier. Therefore, after comprehensively considering the impact of the angle on both the leakage ratio and the size, an angle of 50° is selected for the radial stub. Since the impedance of the diode varies with input power, the LR_1_ under different input power levels is presented in Figure 2d. It can be seen that the BSF maintains the energy leaked to the load from Port A at a level below 3% of the total input energy across a wide range of input power levels, demonstrating the excellent performance of the designed filter.

### 2.2. Low-Pass Filter Design

Building upon the designed BSF, a Chebyshev type-I LPF is selected as the prototype for the LPF within the rectifier. The Chebyshev type-I LPF exhibits a sharper transition between the passband and stopband, achieving significant suppression within a narrow frequency range. The design specifications for the LPF within the rectifier are as follows: pass-band frequency (F_p_) = 2.7 GHz, stop-band frequency (F_s_) = 3.5 GHz, pass-band ripple (A_p_) = 1 dB, and stop-band attenuation (A_s_) = 20 dB. Normalizing frequencies F_p_ and F_s_ with respect to F_p_ yields:

(2) $$\omega_{p}=2\pi F_{p}$$

(3) $$\omega_{s}=\frac{2\pi f_{s}}{\omega_{p}}$$

Substituting ω_s_, A_p_, and A_s_ into the equation:

(4) $$N\geq \frac{\cosh^{-1}\left(\sqrt{\frac{10^{\left(\frac{A_{s}}{10}\right)}-1}{10^{\left(\frac{A_{p}}{10}\right)}-1}}\right)}{\cosh^{-1}\left(\omega_{s}\right)}$$

**Figure 3:**
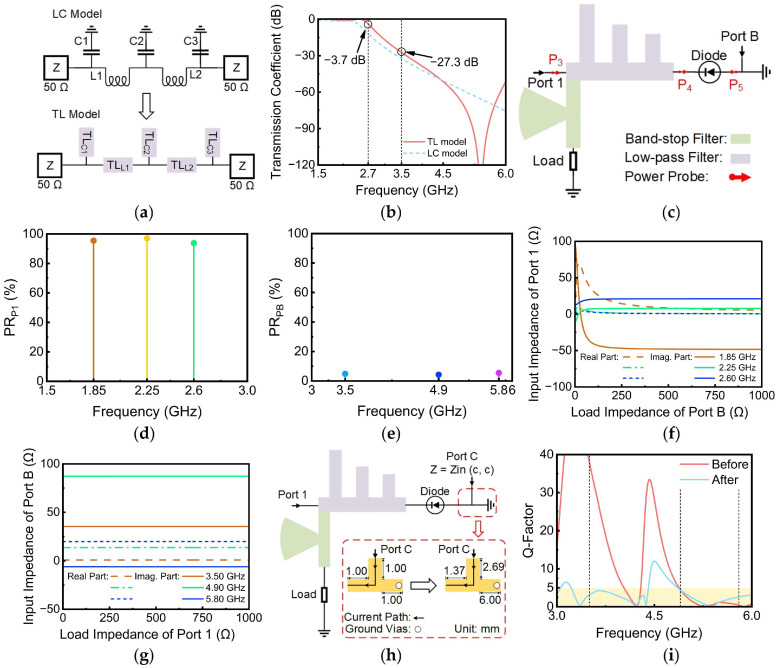
(**a**) LC and TL models of the LPF; (**b**) transmission coefficient of the LPF in the rectifier for the TL model and LC model; (**c**) performance verification circuit of the LPF; (**d**) power passage ratio through the LPF for input at Port 1 (**d**) and Port B (**e**); input impedance variation at the operating frequency of another port versus load impedance at Port 1 (**f**)/Port B (**g**); rectifier ground line optimization: (**h**) line length comparison before and after optimization; (**i**) Q-factor comparison at Port C operating frequency before and after optimization.

According to the calculation from Equation (4), a fifth-order Chebyshev type-I LPF is required to meet the design specifications. Based on the calculation result, an LC model was constructed, as illustrated in Figure 3a. The specific values for each capacitor and inductor in the LC model are provided in Table 1. Since the lumped elements introduce significant insertion losses, the LC model should be converted to a transmission line (TL) model, which exhibits substantially low losses in the microwave band, as shown in Figure 3a. As shown in Figure 3b, the transmission coefficients of both the TL model and the LC model were examined with port input impedances set to 50 Ω. A 20 dB attenuation was achieved before 3.5 GHz, aligning with both the design specifications and theoretical calculations. The results demonstrate that the LC model exhibits higher losses compared to the TL model, corroborating the findings presented earlier.

Although the initial LPF exhibits good performance, its effectiveness may decrease when integrated with the diode and BSF due to complex port impedance that deviates from the standard 50 Ω impedance, as shown in Figure 3c. This discrepancy necessitates a degree of optimization of the LPF to ensure optimal performance and compatibility within the broader circuit architecture. To evaluate the performance of the LPF within the circuit context, Port B and power probes P3–P5 were incorporated into the circuit to monitor the RF current and transmission rate of energy from both input ports at the LPF location. The power passage ratios of the two ports are defined, respectively, as:

(5) $$Passage\;Ratio_{Port\;1}=\frac{\left|Power_{p4}\right|}{Power_{p3}}$$

(6) $$Passage\;Ratio_{Port\;B}=\frac{\left|Power_{p4}\right|}{Power_{p5}}$$

Passage Ratio_Port 1_ (PR_P1_) represents the proportion of energy input from Port 1 that goes into the diode, while Passage Ratio_Port B_ (PR_PB_) represents the proportion of energy input from Port B that goes through the diode. According to the preset RF current path shown in Figure 1, a higher PR_P1_ for a low frequency is desirable, while a lower PR_PB_ for a high frequency is preferable. Taking these two passage ratios as the optimization objective, the LPF can be optimized by ADS software 2020. The performance characteristics of the optimized LPF integrated into the rectifier are illustrated in Figure 3d,e. As depicted in Figure 3d, the PR_P1_ reaches 95% at 1.85 GHz, 2.25 GHz, and 2.6 GHz, indicating excellent transmission efficiency of the LPF at a low frequency. In addition, as illustrated in Figure 3e, the PR_PB_ is only approximately 5% at 3.5 GHz, 4.9 GHz, and 5.8 GHz, demonstrating effective stopband characteristics.

Until now, the LPF has been designed, and one should think about whether one needs to design the MN or the antenna first due to the coupling between two ports. Fortunately, the LPF enables high isolation between Ports 1 and B at higher frequencies, ensuring that the load impedance at one port does not significantly influence the input impedance at the other port within the higher-frequency range. To further investigate this phenomenon, Figure 3f,g illustrate the variation in input impedance at the operating frequency of one port when the load impedance of another port changes. It is observed that when the load impedance of Port B varies, the input impedance at the operating frequency of Port 1 undergoes significant changes, as shown in Figure 3f. Conversely, when the load impedance of Port 1 changes, the input impedance of Port B at the operating frequency remains relatively stable, as depicted in Figure 3g. These findings indicate that the antenna connected to Port 1 does not affect the input impedance of Port B, while the MN connected to Port B causes the input impedance of Port 1 to change. Therefore, the MN should be designed first to determine the unchanged input impedance of Port 1.

Before designing the MN, an analysis of the input impedance at Port C was conducted. This was due to the fact that the quality factor (Q-factor) in a circuit can be viewed as the ratio of the imaginary part of the impedance to the real part [27]. The level of the Q-factor therefore has a direct effect on the effectiveness of multiband matching. A high Q-factor indicates that the circuit exhibits enhanced frequency selectivity within a specific band. This is evidenced by a narrower passband, which enables the band to more accurately align with the desired frequency. However, this also results in greater attenuation for signals at other frequencies, potentially leading to challenges in matching networks across multiple bands. The Q-factors corresponding to the three operating frequencies at this stage are depicted by the red line in Figure 3i. The results indicate that, while the Q-factors for 4.9 GHz and 5.8 GHz are comparatively low, the Q-factor at 3.5 GHz exhibits a notably high magnitude. This high Q-factor at 3.5 GHz introduces significant complexities in the design of the multi-band MN. To mitigate the design complexity for the MN, the grounding configuration within the rectifier was optimized to reduce the Q-factor at 3.5 GHz. Figure 3h illustrates the variations in grounding line lengths before and after optimization. Figure 3i presents a comparative analysis of the Q-factor variations with frequency at Port C before and after optimization. The optimization process yielded minimal changes in the Q-factors at 4.9 GHz and 5.8 GHz, with both remaining below 5. However, a significant reduction in the Q-factor at 3.5 GHz was observed, decreasing from 35 to 4. This reduction in the Q-factor at 3.5 GHz was expected to simplify the subsequent design process of the MN.

### 2.3. Matching Network Design

**Figure 4:**
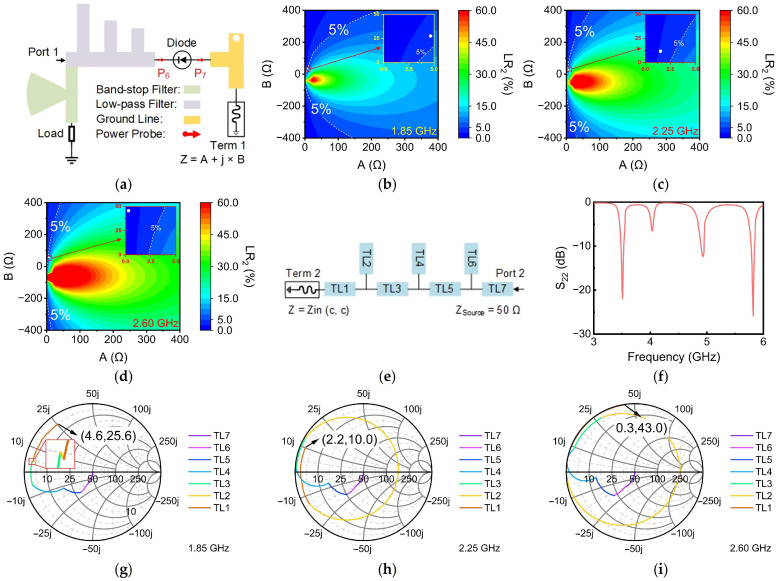
(**a**) Circuit for verifying leakage ratio; power leakage ratio from Port 1 to Port C as a function of Port C impedance at (**b**) 1.85 GHz, (**c**) 2.25 GHz, and (**d**) 2.6 GHz; (**e**) topology of the MN; (**f**) S22 of the rectifier; Smith Chart of impedance variation with the MN at (**g**) 1.85 GHz, (**h**) 2.25 GHz, and (**i**) 2.6 GHz.

Once the LPF has been designed, the design of the MN should be conducted. Before designing the MN, the input impedance at Port C can be extracted. It is worth noting that the function of the MN can be seen as transforming the standard 50 Ω to the conjugate impedance of Port C’s input impedance at 3.5 GHz, 4.9 GHz, and 5.8 GHz. In addition, the MN should also adjust the standard 50 Ω to a desired value at 1.85 GHz, 2.25 GHz, and 2.6 GHz to suppress low-frequency energy transmission from Port 1 to Port C. Therefore, after obtaining the input impedance of Port C, the effect of the load impedance at Port C on the energy leakage is investigated. To perform this study, a variable load termination (named Term 1) was introduced at Port C of the rectifier, as shown in Figure 4a. The impedance of Term 1 was defined as A + j × B, where both A and B can be arbitrarily adjusted. Additionally, two power probes, P6 and P7, were incorporated into the circuit to monitor the magnitude of energy leakage from Port 1 towards Port C. This configuration is illustrated in Figure 4a. The proportion of power leaking from Port 1 to Port C relative to the total input power is defined as:

(7) $$Leakage\;Ratio_{2}=\frac{Power_{p7}}{Power_{p6}}$$

The impact of Term 1’s impedance on Leakage Ratio_2_ (LR_2_) was investigated by simultaneously varying the values of A and B as the frequencies were set as 1.85 GHz, 2.25 GHz, and 2.6 GHz. The resulting trends are presented as contour plots in Figure 4b–d. These figures also highlight the regions where LR_2_ was less than 5%, providing valuable reference data for the design of the MN. This visualization allows for a comprehensive understanding of the relationship between impedance variations and LR_2_, thereby facilitating more informed design decisions.

The design of the MN in this rectifier was completed following the methodology proposed in [28] and is illustrated in Figure 4e. In this configuration, Term 2 with a source impedance of 50 Ω is utilized, and the input impedance of Port C is matched to Term 2 through the MN. By optimizing the MN, the optimum S22 can be obtained, as shown in Figure 4f. The results demonstrate that the S22 at Port 2 remains below −10 dB within the designated input frequency. This indicates excellent matching performance, ensuring efficient power transfer and minimizing reflections at the input port.

To verify the filtering performance of the MN, the impedance variations at 1.85 GHz, 2.25 GHz, and 2.6 GHz with respect to the length of each transmission line segment within the MN were extracted and plotted on Smith charts, as illustrated in Figure 4g–i. As depicted in the figures, the impedance at each frequency began at the 50 Ω source impedance of Port 2 and ultimately reached 4.6 + j × 25.6 Ω, 2.2 + j × 10.0 Ω, and 0.3 + j × 43.0 Ω at 1.85 GHz, 2.25 GHz, and 2.60 GHz, respectively. The final impedances for the three frequencies were identified on the contour plot, magnified locally, and denoted by white points, as illustrated in the enlarged insets of Figure 4b–d. These magnified views reveal that the leakage ratio for all three frequencies was effectively reduced from an initial value exceeding 30% to less than 5%. This significant reduction demonstrates that the MN not only successfully matches the three operating frequencies of Port 2 to 50 Ω, but also simultaneously achieves filtering performance.

**Figure 5:**
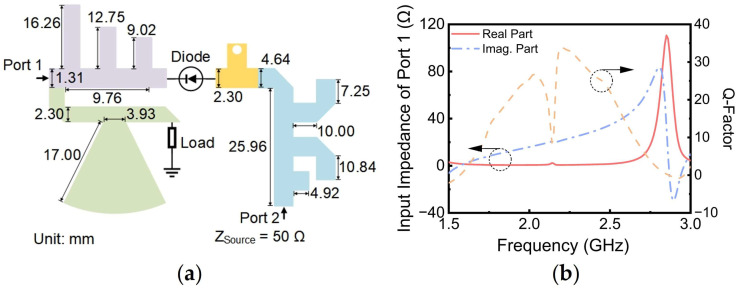
(**a**) Rectifier topology; (**b**) input impedance and Q-factor versus frequency at Port 1 of the rectifier.

Following the completion of the MN design, all structural components were interconnected. The resulting circuit topology, after appropriate folding and optimization, is presented in Figure 5a. This figure also illustrates the dimensions of various sections of the rectifier. The input impedance at Port 1 was then extracted, and its corresponding Q-factor was calculated, as illustrated in Figure 5b. Under these conditions, the real part of the input impedance at Port 1 remained close to 0 Ω within the operating frequency, while the imaginary part exhibited a substantial value. This led to a high Q-factor, hindering the achievement of satisfactory impedance matching. To address this issue, the subsequent design approach eschewed the implementation of an MN for Port 1. Instead, a direct connection between Port 1 and the antenna was established using conjugate matching techniques.

## 3. Antenna Design

The antenna design and optimization were carried out using CST Studio Suite software 2020. The antenna substrate was identical to that of the rectifier, utilizing F4B ($\varepsilon_{r}=2.2,tan\theta =0.001$). The design process began with the development of Antenna 1 (Ant. 1) at Port 1, followed by the design of Antenna 2 (Ant. 2).

### 3.1. Antenna 1

**Figure 6:**
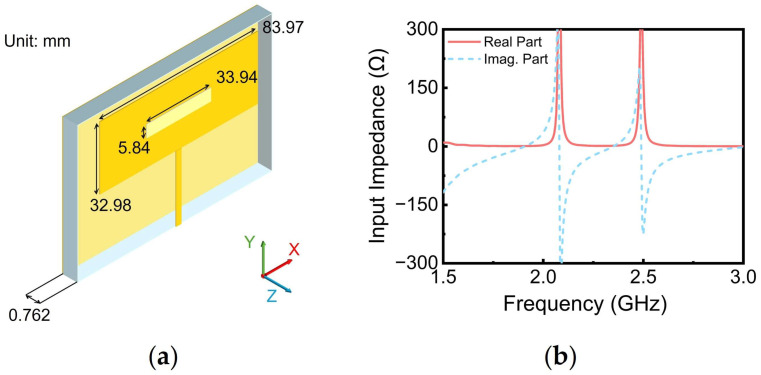
(**a**) Topology of Ant. 1; (**b**) input impedance of Ant. 1.

A simple patch antenna with a rectangular slot etched at its center, as depicted in Figure 6a, was selected as the initial design for Ant. 1. This slot enabled adjustments to the antenna’s input impedance. The input impedance characteristics of Ant. 1 are shown in Figure 6b, revealing two resonant peaks within the frequency range of 2.0–2.5 GHz. Notably, the imaginary part of the input impedance crossed zero near the subsequent resonant peak. This impedance behavior promoted a trend towards conjugate matching between the input impedance of Ant. 1 and that of Port 1 of the rectifier at 1.85, 2.25, and 2.6 GHz. Table 2 presents the input impedances of Antenna 1 and the rectifier’s Port 1 at these frequencies. It is obvious that the input impedances of these two components closely met the conjugate matching requirements, with real parts being approximately equal and imaginary parts exhibiting opposite signs and similar magnitudes.

### 3.2. Antenna 2

**Figure 7:**
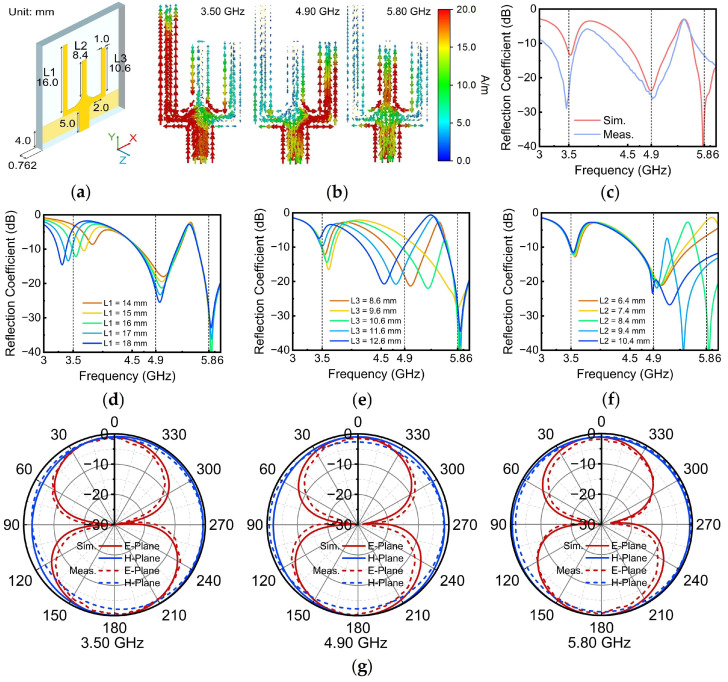
(**a**) Topology of Ant. 2; (**b**) surface current distribution of Ant. 2; (**c**) reflection coefficients of Ant. 2; effect on the resonance of Ant. 2 when the lengths of (**d**) L1, (**e**) L2, and (**f**) L3 are varied; (**g**) radiation patterns.

This section focuses on the design of Ant. 2, which interfaces with Port 2 of the rectifier that has already undergone MN design. It should operate at three distinct frequencies: 3.5 GHz, 4.9 GHz, and 5.8 GHz. To achieve multi-band functionality, three individual monopole antennas, each resonant at one of the target frequencies, were combined and strategically bent to form the desired antenna structure, as shown in Figure 7a. Each branch of Ant. 2 corresponded to a specific resonant frequency: the left branch at 3.5 GHz, the middle branch at 4.9 GHz, and the right branch at 5.8 GHz. This is confirmed by the surface current distribution depicted in Figure 7b, which shows that at 3.5 GHz, the current was predominantly concentrated on the left branch; at 4.9 GHz, the current was primarily on the middle branch; and at 5.8 GHz, the current was mainly on the right branch. Figure 7c presents the simulated and measured reflection coefficients of Ant. 2. The measurement results indicate that the operating bands of Ant. 2 aligned with those demonstrated in the simulation. However, there was a discrepancy in the reflection coefficient, which may be attributed to the impact of SMA connector soldering on antenna performance during fabrication, as well as errors introduced by the testing environment. This is particularly relevant because an omnidirectional radiation pattern places high demands on the testing environment. Nevertheless, the measurement results demonstrate that the reflection coefficient of Ant. 2 remained below −10 dB for all three operating bands, satisfying the requirement for connection to the rectifier. The effect of the length of the stubs used in the antenna design on the resonance of the antenna was also investigated. It can be seen from Figure 7d that as L1 increased, the first resonant frequency shifted to a lower frequency. In addition, similar trends can be found in Figure 7e,f: as L3 and L2 increased, the second and third resonant frequencies shifted to lower frequencies. Therefore, the optimum reflection coefficient could be obtained by adjusting the lengths of the three stubs. Additionally, the simulated and measured radiation patterns of Ant. 2, as shown in Figure 7g, demonstrated desirable omnidirectional characteristics in the H-plane and a wide beamwidth in the E-plane across all operating frequencies.

## 4. Six-Band Rectenna

**Figure 8:**
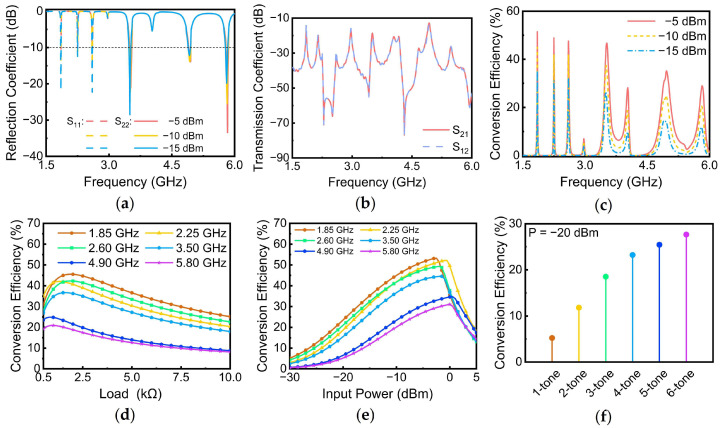
(**a**) Reflection coefficient of the rectenna at different input power levels in simulations; (**b**) transmission coefficient of the rectenna in simulations; (**c**) PCE of the rectenna at different input power levels in simulations; (**d**) PCE versus load resistance for each operating frequency in simulations; (**e**) PCE versus input power for each operating frequency in simulations; (**f**) PCE of the rectenna for one to six input tones at an input power of −20 dBm in simulations.

Once the antennas were designed, a complete six-band dual-port rectenna was realized by integrating Ant. 1 and Ant. 2 with the designed rectifier. The simulated reflection coefficients of the rectenna are shown in Figure 8a. Both ports of the rectenna exhibited good matching (S11 and S22 < −10 dB) across a range of input power levels (−5 dBm, −10 dBm, and −15 dBm), demonstrating the rectenna’s ability to maintain its operating state over a wide input power range. The simulated transmission coefficients between the two ports of the rectenna are presented in Figure 8b. It can be seen that the S12 and S21 were lower than −15 dB at six frequency bands, indicating good isolation between the two ports. In addition, the PCE of the rectenna as a function of frequency under different input power levels is presented in Figure 8c. At an input power of −10 dBm, the PCE reached 45.0% at 1.85 GHz, 41.9% at 2.25 GHz, 41.8% at 2.6 GHz, 36.7% at 3.5 GHz, 23.4% at 4.9 GHz, and 20.1% at 5.8 GHz, respectively.

To determine the optimal load resistance for the rectenna, Figure 8d illustrates the PCE variation as a function of load resistance across each operating frequency band. The PCE in each band exhibited a trend of initial increase followed by a decrease with increasing load resistance. To balance the PCE across all operating bands, a load resistance of 1500 Ω was selected for the rectenna. In addition, to investigate the effect of the input on the PCE, the PCE as a function of input power at six frequencies is presented in Figure 8e. It is obvious that the PCE increased gradually as the input power rose. However, once the input power reached a certain threshold (−2.5 dBm at 1.85 GHz, −0.5 dBm at 2.25 GHz, −1.5 dBm at 2.6 GHz, −1.5 dBm at 3.5 GHz, 0.5 dBm at 4.9 GHz, and 0 dBm at 5.8 GHz), the PCE began to decline rapidly. This phenomenon occurred because as the input power increased, the rectifier converted more DC energy. Once the energy exceeded the reverse breakdown voltage of the diode, a significant reverse breakdown current was generated within the diode. This current flowed through the diode, causing it to heat up and resulting in substantial energy loss in the form of heat rather than being delivered to the load. Consequently, this led to a decrease in conversion efficiency.

Finally, Figure 8f illustrates the PCE of the rectenna under one to six tone input conditions. The 5.8 GHz was selected as the initial single-tone input, and additional frequency bands were progressively incorporated in descending order of frequency. At an ultra-low input power level of −20 dBm, the rectenna’s PCE demonstrated a significant improvement, rising from 5.2% for single-tone input to 27.6% for multi-tone input, representing a substantial enhancement of 22.4%. This marked improvement effectively enhanced the rectenna’s performance under ultra-low input power conditions.

**Figure 9:**
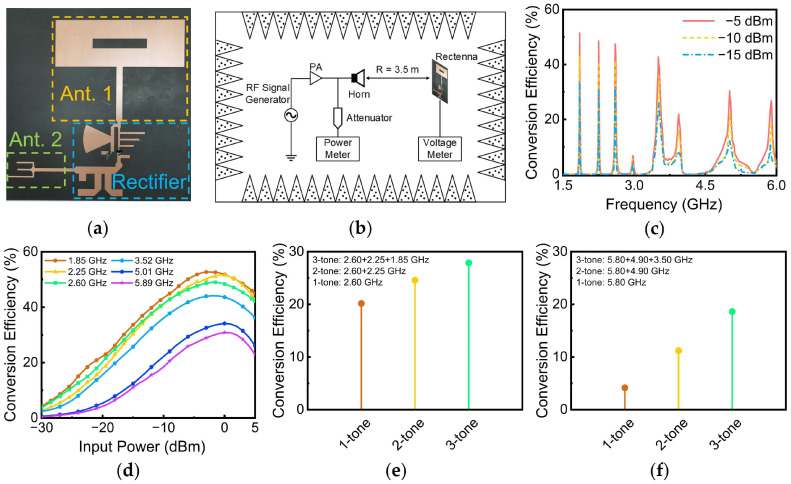
(**a**) Photograph of the rectenna; (**b**) test environment setup; (**c**) PCE of the rectenna at different input power levels during measurements; (**d**) PCE versus input power for each operating frequency during measurements; PCE for one to three input tones during measurements: (**e**) Ant. 1, (**f**) Ant. 2.

To validate the performance of the rectenna under practical conditions, a physical prototype of the rectenna was fabricated, as illustrated in Figure 9a. To measure the PCE of the rectenna, a horn antenna connected to an RF signal generator and a power amplifier was used to transmit RF energy, as shown in Figure 9b. According to the Friis transmission equation, the power received by the rectenna could be calculated using the following equation:

(8) $$P_{r}=P_{t}+G_{r}+G_{t}+20\;\log_{10}\left(\frac{\lambda }{4\pi R}\right)$$

where P_t_ and G_t_ represent the transmitted power and gain of the horn antenna, respectively; G_r_ denotes the gain of the rectenna; and R is the distance between the two antennas, taken as 3.5 m.

Figure 9c presents the measured PCE of the rectenna as a function of frequency at different input power levels. At an input power of −10 dBm, the PCE values for the rectenna were 43.01% @ 1.85 GHz, 41.00% @ 2.25 GHz, 41.33% @ 2.6 GHz, 35.88% @ 3.52 GHz, 22.36% @ 5.01 GHz, and 19.27% @ 5.89 GHz. Notably, the operating frequencies for the branch containing Ant. 2 exhibited some deviation, which can be attributed to manufacturing errors introduced during fabrication.

Figure 9d illustrates that the measured PCE of the rectenna varied with the input power at six frequency bands. Similar to the simulation results, the PCE initially increased gradually with rising input power, and then began to decline once the input power reached a certain threshold. However, the rate of decline was comparatively slower than that observed in the simulation results.

Due to limitations in the testing conditions, the multi-tone PCE test was performed for a low frequency and a high frequency, respectively. As shown in Figure 9e, the multi-tone PCE for the low frequency increased from 20.17% at a single frequency (2.6 GHz) to 27.88% at three frequencies. Similarly, the multi-tone PCE for the high frequency improved from 4.17% at a single frequency (5.8 GHz) to 18.64% at three frequencies, as shown in Figure 9f. Both measured results demonstrate an enhancement in PCE under multi-tone input compared to single-tone input, with the multi-tone PCE for the high frequency achieving an increase of 14.47%.

Finally, a comparison between our design and other related designs is presented in Table 3. It is obvious that our design achieved a rectenna capable of simultaneous operation at six frequencies, using fewer diodes and, consequently, fewer rectifying branches. Moreover, at an input power of −10 dBm, our design attained a higher conversion efficiency compared to other works. Although the operating frequency bands were relatively fewer compared with those in [15], the proposed design achieved higher PCE than that reported in [15]. Moreover, compared with the design presented in [19], the proposed design demonstrated a more substantial improvement in RF-to-DC conversion efficiency under multi-tone input conditions. These improvements can be primarily attributed to the reduced losses achieved through the implementation of a single-diode configuration. Therefore, the rectenna proposed in this work offers certain advantages in terms of the number of simultaneously operable frequencies and conversion efficiency when compared to similar rectenna designs in other studies.

## 5. Conclusions

An innovative six-band, dual-port rectenna was proposed and fabricated, combining two half-wave rectification methods. This design allows for the independent optimization of each structure without compromising the others. The experimental results demonstrate that the rectenna maintains high PCE even at ultra-low input power levels. Moreover, when subjected to simultaneous multi-frequency inputs, the rectenna exhibits enhanced overall PCE. The proposed rectenna operates efficiently across the 4G, 5G, and WLAN frequency bands, making it particularly suitable for powering low-power devices, such as sensors in IoE scenarios. These scenarios include networked devices in smart homes and consumer mobile devices. This capability addresses the increasing demand for energy-efficient solutions in various wireless applications.

## Figures and Tables

**Table 1:** Specific parameters of each capacitor and inductor in the LC model.

Name of Capacitor	Capacitance (pF)	Name of Inductor	Inductance (nH)
C1	2.52	L1	3.22
C2	3.54	L2	3.22
C3	2.52		

**Table 2:** Input impedance of the rectifier (Port 1) and Ant. 1.

	1.85 GHz	2.25 GHz	2.6 GHz
Conjugate Antenna	0.8 − j × 11.49	1.5 − j × 23.3	3.0 − j × 42.2
Port 1 of Rectifier	0.6 + j × 11.45	0.7 + j × 23.3	3.0 + j × 42.2

**Table 3:** Comparison of this work with referenced works.

Ref.	Number of Bands	Freq. (GHz)	Forms of the Rectifier	Number of Diodes	PCE @ −10 dBm (%)	Enhancement of the PCE Under Multi-Tone Input @ −20 dBm (%)	Output Voltage @ −10 dBm (V)
[14]	3	0.85/1.77/2.07	single MN	1	50/40/35	NR *	NR *
[15]	8	0.84/1.29/1.68/3.08/3.45/4.31/5.11/5.49	single MN	1	52/53/25/25/34/14/3/13	NR *	0.51/0.5/0.39/0.4/0.45/0.25/0.18/0.27
[19]	5	0.59/0.77/0.92/1.85/2.32	stacked	6	47/42/50/37/39	18	NR *
[29]	4	0.9/1.8/2.45/5.8	single MN	1	7/9/11/2	NR *	NR *
[30]	4	0.84/1.86/2.1/2.45	stacked	4	19/15/21/7	NR *	NR *
[31]	3	0.9/1.8/2.45	stacked	6	34/22/20	NR *	0.28/0.26/0.25
**This Work**	**6**	**1.85/2.25/2.6/3.52/5.01/5.89**	**hybrid**	**1**	**43/41/41/36/22/19**	**22.5**	**0.26/0.25/0.25/0.23/0.18/0.17**

* NR: not reported.

## Data Availability

The data presented in this study are available on request from the corresponding author.
